# Structural characterisation reveals insights into substrate recognition by the glutamine transporter ASCT2/SLC1A5

**DOI:** 10.1038/s41467-017-02444-w

**Published:** 2018-01-02

**Authors:** Amanda J Scopelliti, Josep Font, Robert J Vandenberg, Olga Boudker, Renae M Ryan

**Affiliations:** 1 Transporter Biology Group, Discipline of Pharmacology, Sydney Medical School, University of Sydney, Sydney, NSW 2006 Australia; 2 Department of Physiology and Biophysics, Weill Cornell Medicine, New York, NY 10065 USA; 3 Howard Hughes Medical Institute, Weill Cornell Medicine, New York, NY 10065 USA

## Abstract

Cancer cells undergo a shift in metabolism where they become reliant on nutrients such as the amino-acid glutamine. Glutamine enters the cell via the alanine/serine/cysteine transporter 2 (ASCT2) that is upregulated in several cancers to maintain an increased supply of this nutrient and are therefore an attractive target in cancer therapeutic development. ASCT2 belongs to the glutamate transporter (SLC1A) family but is the only transporter in this family able to transport glutamine. The structural basis for glutamine selectivity of ASCT2 is unknown. Here, we identify two amino-acid residues in the substrate-binding site that are responsible for conferring glutamine selectivity. We introduce corresponding mutations into a prokaryotic homologue of ASCT2 and solve four crystal structures, which reveal the structural basis for neutral amino acid and inhibitor binding in this family. This structural model of ASCT2 may provide a basis for future development of selective ASCT2 inhibitors to treat glutamine-dependent cancers.

## Abstract (web summary)

Cancer cells are reliant on nutrients such as glutamine, which enter the cell via the alanine/serine/cysteine transporter 2 (ASCT2). Here, authors use crystallography to show which amino-acid residues in the substrate-binding site are responsible for conferring glutamine selectivity to ASCT2.

## Introduction

Cancer cells are known to undergo a metabolic shift toward glycolysis, resulting in an increased reliance on nutrients, such as the amino-acid glutamine^1,2^. Glutamine enters the cell via membrane-bound transporters such as the alanine, serine, cysteine transporter 2 (ASCT2/SLC1A5) and provides an alternate fuel source for the tricarboxylic acid cycle, contributes to the activation of the mammalian target of rapamycin complex 1 (mTORC1) and is a source of fatty-acid production^3^. ASCT2 expression is upregulated in several cancer types, including glioma, melanoma, prostate, colon, hepatoma and breast cancer^4–10^. ASCT2 is the predominant glutamine transport system in several cancers^11–13^, where glutamine becomes an essential amino acid required to fuel growth and proliferation^14^. Therefore, selective inhibitors of ASCT2 show promise as novel anticancer therapeutics^12,15^.

ASCT2 belongs to the solute carrier family 1A (SLC1A), which contains membrane transporters for acidic and neutral amino acids including the excitatory amino-acid transporters (EAATs) and the prokaryotic aspartate transporters Glt_Ph_^16–19^ and Glt_Tk_^20^. ASCT1 (SLC1A4) and ASCT2 (SLC1A5) are sodium (Na^+^)-dependent neutral amino-acid transporters found throughout the body where they regulate neutral amino-acid pools. Both transport small neutral amino acids including alanine, serine, cysteine and threonine^16,17,21^, whereas ASCT2 also transports glutamine, asparagine, methionine, glycine and leucine^18^. ASCT1 and ASCT2 share ~ 57% amino-acid sequence identity^16–18^, however the molecular basis for the differences in substrate selectivity between them, and in particular, how ASCT2 is able to support glutamine transport is unknown. Current inhibitors of ASCT2 include non-selective compounds such as serine biphenyl-4-carboxylate^22^, benzylserine^23^ and the glutamine-based inhibitor, glutamyl-p-nitroanilide (GPNA)^24^. More recently, derivatives of GPNA, including 2-Amino-4-bis(aryloxybenzyl)aminobutanoic acid, and benzylproline have been developed but the selectivity of these inhibitors for ASCT2 over ASCT1, or other glutamine transporters has not been established^25–27^. A detailed understanding of the molecular basis for substrate selectivity of the SLC1A family is crucial for the development of high-affinity and selective ASCT2 compounds.

**Fig. 1:**
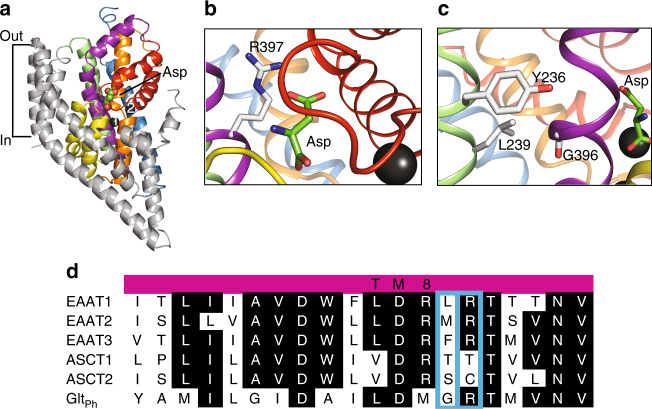
The structure of a Glt_Ph_ protomer, and sequence alignments of ASCTs and EAATs and Glt_Ph_ **a** Glt_Ph_ protomer (PDB: 2NWX) shown in the plane of the membrane, with the trimerization domain (TM1, 2, 4 and 5) in grey and the transport domain: TM3 (blue), TM6 (green), TM7 (orange), TM8 (purple), HP1 (yellow) and HP2 (red). L-Aspartate and two bound Na^+^ ions (dark grey spheres) are shown. Close-up view of the substrate-binding site is shown with **b** R397 and **c** Y236, L239 and G396 shown in stick representation. In **c**, the protein has been rotated for better visualisation of the TM3 and TM6 interface. Images were made using Pymol^64^. **d** Sequence alignment of part of TM8 in EAAT1-3, ASCT1-2 and Glt_Ph_, where conserved residues are highlighted in black, and mutated residues are highlighted by blue boxes

ASCTs share ~ 23% amino-acid sequence identity with Glt_Ph_^28^ and ~ 40% amino-acid sequence identity with the EAATs^29,30^. Neutral amino-acid exchange via the ASCTs is thought to require binding of three Na^+^ ions^21,31–33^ whereas acidic amino-acid transport by the EAATs is coupled to the co-transport of three Na^+^ ions, one proton (H^+^) and is followed by the counter-transport of one potassium (K^+^) ion^34,35^. The binding of Na^+^ and substrate to both the ASCTs and the EAATs also activates a thermodynamically uncoupled anion conductance^21,29,31^. A prokaryotic cousin of the ASCTs and the EAATs, the aspartate transporter Glt_Ph_ from an archaeon *Pyrococcus horikoshii*, was first crystallised in 2004, revealing the structure of the SLC1 transporter family^28^. Glt_Ph_ is a Na^+^-dependent transporter that is selective for aspartate. Like the EAATs and ASCTs, Glt_Ph_ is also coupled to the co-transport of three Na^+^ ions and contains an uncoupled anion conductance^36–38^. It exists as a homotrimer with each protomer containing 8 transmembrane domains (TM1-8) and 2 hairpin loops (HP1, 2) (Fig. 1a). An additional crystal structure of Glt_Ph_ revealed the binding sites for aspartate and two of the three coupled Na^+^ ions^39^ (Fig. 1). The location of the third Na^+^ ion, predicted based on computational and mutagenesis studies^40^, has recently been observed in the structure of another closely related archaeal homologue Glt_Tk_^20^. In addition, a crystal structure of a human thermally stabilised mutant of EAAT1 bound to aspartate pictured a substrate-binding site nearly identical to that of Glt_Ph_^41^. In the substrate-binding site, an arginine residue in TM8, conserved throughout the acidic amino-acid transporters in the SLC1 family, interacts with the carboxylate group of the substrate aspartate in Glt_Ph_ (R397; Fig. 1b) and in human EAAT1 (R459)^41^. When this residue is mutated to a cysteine or threonine in human or mouse EAAT1, respectively, concentrative acidic amino-acid transport is abolished^42,43^ whereas neutral amino-acid exchange is introduced^44^. The equivalent residue in ASCT1 is a threonine (T459; Fig. 1d), and we have shown that mutation of this residue to arginine alters the substrate specificity of ASCT1 to prefer acidic amino acids^45^. The residue equivalent to R397 in ASCT2 is a cysteine (C482; Fig. 1d). The role of this cysteine residue in substrate binding to ASCT2 has not been investigated.

To explore the molecular basis for substrate selectivity of the ASCTs, we target T459 of ASCT1 and C482 of ASCT2 for mutagenesis, where each residue was replaced with the corresponding residue in the other ASCT subtype (T459C in ASCT1 and C482T in ASCT2). An additional mutation was also introduced at the preceding residue using the same approach (T458S in ASCT1 and S481T in ASCT2). The substrate selectivity profiles of ASCT1 and ASCT2 transporters are switched as a result of these mutations, where L-glutamine exchange is introduced into ASCT1, and abolished in ASCT2. Similarly, mutating the equivalent residues in Glt_Ph_ to their ASCT2 counterparts (G396S and R397C) switches the substrate selectivity from acidic to neutral amino acids, where transport and/or binding of a variety of neutral amino-acid substrates and inhibitors is observed. We solve four structures of Glt_Ph_-R397C revealing the structural basis for neutral amino-acid substrate and inhibitor binding to ASCT2. Our results provide insights into the structural basis for substrate selectivity in the SLC1A family and we present a prokaryotic model of neutral amino-acid transport that may be utilised for structure-based ASCT2 inhibitor development.

## Results

### Characterisation of wild-type ASCT1 and ASCT2

**Fig. 2:**
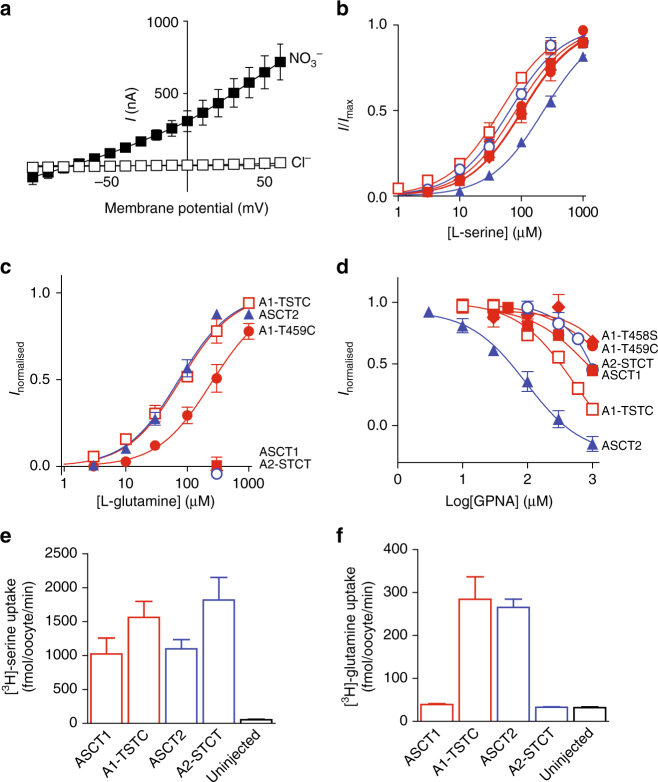
Mutations in ASCT1 and ASCT2 affect substrate and inhibitor selectivity **a** Current–voltage relationships elicited by 300 µM L-serine in Cl^−^-containing buffer (open squares) and NO_3_^−^-containing buffer (closed squares), at pH 7.5 in wild-type ASCT1. Concentration–response curves are shown for L-serine **b** and L-glutamine **c** in ASCT1 (red, closed squares), ASCT2 (blue, closed triangles), A1-T458S (red diamonds), A1-T459C (red, closed circles), A1-T458S/T459C (red, open squares) and A2-S481T/C482T (blue, open circles) at+60 mV. As L-glutamine concentration–response curves for ASCT1, A1-T458S and A2-S481T/C482T was not determined, data were normalised to the predicted maximal current for L-serine for each transporter. **d** Concentration–response curves are shown for GPNA in ASCT1 (red, closed squares), ASCT2 (blue, closed triangles), A1-T459C (red, closed circles), A1-T458S (red diamonds), A1-T458S/T459C (red, open squares) and A2-S481T/C482T (blue, open circles). GPNA was co-applied with an EC_50_ value of L-serine for the respective transporters and normalised to the current generated by the application of L-serine alone. L-[^3^H]serine **e** and L-[^3^H]glutamine **f** uptake into oocytes expressing wild-type and mutant ASCT1 (red), ASCT2 (blue) transporters and uninjected control oocytes (black). Oocytes were incubated in Cl^−^-containing buffer with 10 µM L-[^3^H]substrate at room temperature, pH 7.5 for 10 min. Values presented are mean ± S.E.M, (*n* ≥ 3)

The exchange of neutral amino acids by ASCT1 and ASCT2 is electroneutral. However, the binding of substrate and Na^+^ activates an uncoupled anion conductance mediated by the transporters that follows the permeability sequence of nitrate (NO_3_^−^) > iodide (I^−^) > bromide (Br^−^) > chloride (Cl^−^). In buffers containing NO_3_^−^, application of substrate to oocytes expressing ASCT1 or ASCT2 generates robust currents that reverse direction at approximately −60 mV (Fig. 2a). Application of increasing doses of L-serine were applied yielding EC_50_’s of L-serine for ASCT1 (100 ± 20 µM; S.E.M.; *n* ≥ 3) and ASCT2 (230 ± 30 µM; *n* ≥ 3) (Fig. 2b; Table 1). Similarly, we observe L-[^3^H]serine uptake into oocytes expressing ASCT1 and ASCT2 (Fig. 2e). Application of L-glutamine generates outward currents when applied to oocytes expressing ASCT2 with an EC_50_ of 80 ± 10 µM; *n* ≥ 3, but not when applied to ASCT1 (Fig. 2c; Table 1). In addition, oocytes expressing ASCT2 display robust L-[^3^H]glutamine uptake while no glutamine transport is observed for oocytes expressing ASCT1 (Fig. 2f). Application of 300 µM GPNA, a previously identified ASCT2 inhibitor^24^, inhibits the L-serine activated current of ASCT2 by 90 ± 5% with an IC_50_ of 90 ± 20 µM; *n* ≥ 3 compared with 26 ± 3% inhibition of ASCT1 (Fig. 2d; Table 1; Supplementary Figure 1). Thus, both glutamine and the glutamine-based inhibitor GPNA show a preference for ASCT2 over ASCT1.

**Table 1:** Altered substrate and inhibitor selectivity of mutant ASCT1 and ASCT2 transporters

	ASCT1	A1-T459C	A1-T458S	A1-T458S/T459C	ASCT2	A2-S481T/S482C
L-Ser EC_50_ (μM)	100 ± 20	83 ± 6	106 ± 4	45 ± 5	250 ± 40	61 ± 7
L-Gln EC_50_ (μM)	–	260 ± 80	–	90 ± 10	80 ± 10	–
GPNA % inhibition	26 ± 3	18 ± 1	ND	41 ± 4	90 ± 5	20 ± 3

To establish EC_50_s, varying concentrations of L-serine or L-glutamine was applied in a NO_3_^−^-containing buffer, and the current (I) at + 60 mV measured. EC_50_ values of L-serine were competed against 1 mM GPNA to determine the level of inhibition. Currents generated in the presence of inhibitor were subtracted from currents generated in the absence of inhibitor to determine % inhibition. All values represent mean ± S.E.M (*n* ≥ 3). A dash indicates the substrate did not generate currents; ND indicates the value was not determined.

### Mutations in ASCT1

To probe the molecular basis for differences in substrate selectivity between ASCT1 and ASCT2 we mutated two residues in TM8 of both transporters. The crystal structure of Glt_Ph_ and EAAT1 in complex with substrate reveals a direct interaction between bound aspartate and R397 in TM8 (R459 in EAAT1, Fig. 1b)^39,41^. Mutations of the equivalent residue in the EAATs and ASCT1 can switch the substrate selectivity between acidic and neutral amino acids^44,45^. The equivalent residue is not conserved between ASCT1 and ASCT2. Therefore, we hypothesised that mutation of the residue in ASCT1 (Thr459) to the ASCT2 counterpart (Cys) may alter substrate selectivity. Indeed, the mutant ASCT1 transporter, A1-T459C generates outward currents in response to both L-serine and L-glutamine application, with apparent affinities of 83 ± 6 µM; *n* ≥ 3 for L-serine (Fig. 2b), and 260 ± 80 µM; *n* ≥ 3 for L-glutamine (Fig. 2c; Table 1). Thus, the subtle change from threonine to cysteine is able to introduce L-glutamine-binding into ASCT1. In contrast, a maximal dose of the inhibitor, GPNA only reduces L-serine activated currents of A1-T459C by 18 ± 1%, which is comparable to that of wild-type ASCT1 (Fig. 2d; Table 1), suggesting that further changes are required for effective binding of the bulkier inhibitor, GPNA.

There are no other candidate residues within the substrate-binding pocket that may contribute to substrate or inhibitor selectivity in the ASCTs. Interestingly, the residue preceding R397 in Glt_Ph,_ G396 is positioned on the back of TM8 away from the substrate-binding site and towards TM6 where it may pack against Y236 and L239 in the available crystal structures (Fig. 1c). G396 is not conserved among the SLC1A family; it is a leucine in EAAT1, a threonine in ASCT1 and a serine in ASCT2 (Fig. 1d). We hypothesised that the variation of this residue could influence the positioning of TM8, and therefore the size and/or conformation of the substrate-binding pocket. We mutated the equivalent residue in ASCT1 (T458) to serine, which is found in ASCT2. Similarly to wild-type ASCT1, A1-T458S generates outward currents in response to L-serine but does not respond to L-glutamine (Fig. 2c), nor exhibit increased sensitivity to GPNA inhibition (Fig. 2d). However, when we introduced both adjacent mutations into ASCT1 to generate the double mutant transporter A1-T458S/T459C, outward currents were observed in response to both L-serine and L-glutamine with increased apparent affinities compared with that of A1-T459C alone (EC_50_ for L-serine is 45 ± 5 µM; *n* ≥ 3 and L-glutamine is 80 ± 10 µM; *n* ≥ 3; Fig. 2c, d; Table 1). Oocytes expressing A1-T458S/T459C show robust accumulation of L-[^3^H]serine and L-[^3^H]glutamine similar to wild-type ASCT1 and ASCT2, respectively (Fig. 2e, f) confirming that the double mutant is a functional serine and glutamine transporter. A1-T458S/T459C is also more sensitive to GPNA, which inhibits L-serine activated currents by 41 ± 4% at 300 µM with an IC_50_ of 600 ± 200 µM; *n* ≥ 3 (Fig. 2d; Table 1). Thus, the removal of a single methyl group bordering the binding pocket increases the affinity of A1-T458S/T459C for both glutamine and GPNA, generating a transporter that more closely resembles ASCT2.

### Mutations in ASCT2

We conducted reverse mutagenesis, replacing S481 and C482 in ASCT2 with their ASCT1 counterparts (both threonine residues). Application of L-serine to oocytes expressing the double mutant transporter (A2-S481T/C482T) generates outward currents with an EC_50_ of 61 ± 7 µM (Fig. 2c; Table 1; *n* ≥ 3), however application of L-glutamine did not generate any currents (Fig. 2c). Similarly, A2-S481T/C482T displays uptake of L-[^3^H]serine (Fig. 2e), but not L-[^3^H]glutamine (Fig. 2f). The sensitivity of A2-S481T/C482T to GPNA was reduced compared with wild-type ASCT2, and closely resembled that of wild-type ASCT1 (Fig. 2d; Table 1). These results demonstrate that S481 and C482 play a crucial role in the binding of L-glutamine and the L-glutamine-derivative GPNA to ASCT2, revealing the subtle structural differences that differentiate substrate selectivity between ASCT1 and ASCT2.

### Mutations in Glt_Ph_

**Fig. 3:**
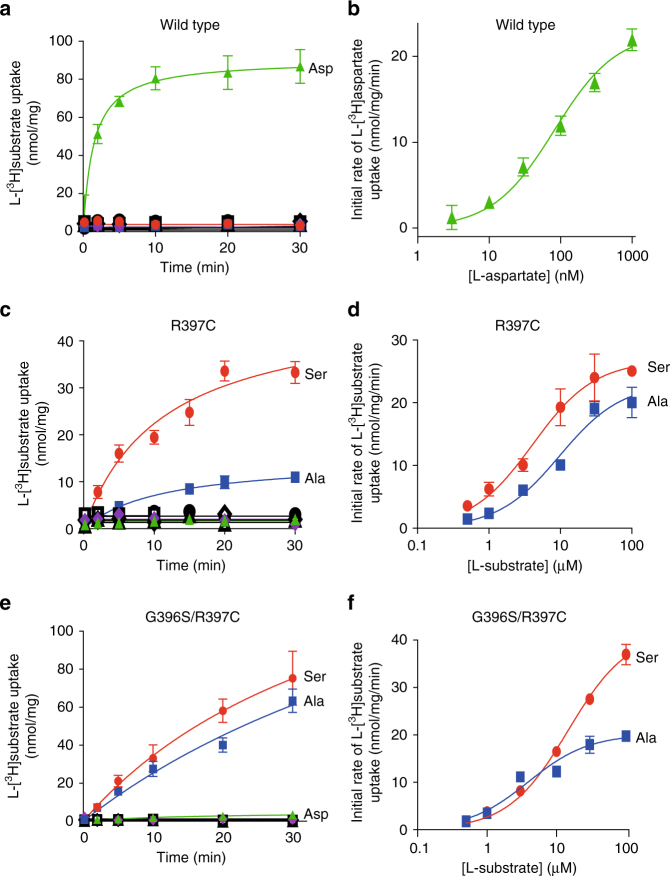
Mutations in Glt_Ph_ alter the substrate selectivity to prefer neutral amino acids L-[^3^H]aspartate (100 nM; green triangles), L-[^3^H]serine (1 µM; red circles), L-[^3^H]alanine (1 µM; blue squares) and L-[^3^H]glutamine (1 µM; purple diamonds) transport by wild-type Glt_Ph_ **a**, Glt_Ph_-R397C **c** and Glt_Ph_-G396S/R397C **e** in the presence of an inwardly directed Na^+^ gradient at pH 7.5. Uptake of each substrate in the absence of a Na^+^ gradient is shown in open, black symbols, of which multiple are overlayed. **b** L-[^3^H]aspartate concentration–response for wild-type Glt_Ph_. L-[^3^H]serine (red circles) and L-[^3^H]alanine (blue squares) concentration responses are shown for Glt_Ph_-R397C **d** and Glt_Ph_-G396S/R397C **f**. Values presented are mean ± S.E.M, (*n* ≥ 3)

Multiple crystal structures of Glt_Ph_ have been solved and provide information about the various stages of the transport cycle of SLC1A family members^39,46,47^. However, structural information regarding neutral amino-acid transport via the ASCTs is limited. Our aim was to generate a more accurate model of neutral amino-acid transport by altering the substrate selectivity of Glt_Ph_ to reflect that of ASCT2 via mutagenesis. Purified Glt_Ph_ variants were reconstituted into liposomes and transport was analysed by measuring L-[^3^H]substrate uptake. In the presence of an inwardly directed Na^+^ gradient, liposomes containing wild-type Glt_Ph_ display robust L-[^3^H]aspartate uptake (Fig. 3a), with an EC_50_ of 82 ± 16 nM (Fig. 3b; *n* ≥ 3). L-[^3^H]serine, L-[^3^H]alanine and L-[^3^H]glutamine are not transported by wild-type Glt_Ph_ (Fig. 3a; *n* ≥ 3).

The two mutations that permit glutamine transport activity in ASCT1 were introduced into Glt_Ph_ (G396S and R397C) (see Figs. 1d and 2) and were well tolerated (Supplementary Figure 2a, b). The single mutant, Glt_Ph_-R397C is no longer capable of transporting L-[^3^H]aspartate under our experimental conditions (Fig. 3c). However, Glt_Ph_-R397C effectively transports L-[^3^H]serine (EC_50_, 4.5 ± 0.9 µM; Fig. 3c, d; *n* ≥ 3) and L-[^3^H]alanine, although to a lesser extent (EC_50_, 14 ± 3 µM; Fig. 3c,d; *n* ≥ 3). Conversely, L-[^3^H]glutamine is not transported at levels above background (Fig. 3c). The double mutant transporter Glt_Ph_-G396S/R397C does not effectively transport L-[^3^H]aspartate or L-[^3^H]glutamine (Fig. 3e), but does display robust L-[^3^H]serine and L-[^3^H]alanine transport (EC_50_’s, 15 ± 2 µM and 4.0 ± 0.2 µM, respectively; Fig. 3e,f; *n* ≥ 3). In the absence of the K^+^-ionophore valinomycin, transport of L-[^3^H]serine is reduced by > 50% for both Glt_Ph_-R397C and Glt_Ph_-R397C/G396S (Supplementary Figure 2c). These results indicate that neutral amino-acid transport by Glt_Ph_-R397C and Glt_Ph_-R397C/G396S is concentrative and electrogenic in contrast to ASCT1 and ASCT2, that function as Na^+^-dependent electroneutral exchangers and are unable to perform concentrative transport^21,31^.

**Fig. 4:**
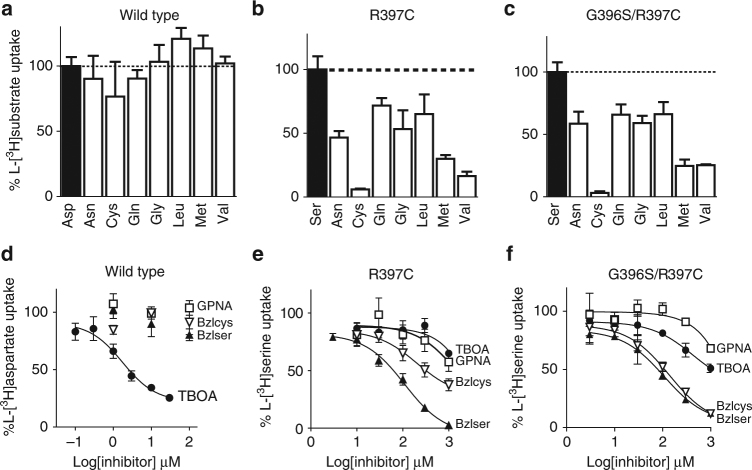
Neutral amino acid and inhibitor selectivity in wild-type and mutant Glt_Ph_ Initial rates of uptake in the presence of 100 nM L-[^3^H]aspartate (black bar) for wild type **a** or 10 µM L-[^3^H]serine (black bars) for Glt_Ph_-R397C **b** and Glt_Ph_-G396S/R397C **c** and 100-fold unlabelled competitor as indicated on the *x*-axis (white bars). Values are normalised to initial rates of L-[^3^H]substrate uptake alone, *n* = 3 ± SEM. Concentration–response curves are shown for TBOA (closed circles), benzylserine (closed triangles), benzylcysteine (open triangles) and GPNA (open squares) in wild-type Glt_Ph_ **d**, Glt_Ph_-R397C **e** and Glt_Ph_-G396S/R397C **f**. Values shown represent mean ± SEM, *n* ≥ 3

To determine the substrate selectivity profile of the mutant Glt_Ph_ transporters, a competition assay was performed where L-[^3^H]aspartate or L-[^3^H]serine uptake was measured in the presence of 100-fold excess unlabelled amino acids. These include cysteine and several amino acids that are known substrates of ASCT2 (asparagine, glutamine, glycine, leucine, methionine and valine) but are not transported by ASCT1^18,37^. L-[^3^H]aspartate transport by wild-type Glt_Ph_ is not inhibited by 100-fold excess of the neutral amino acids tested (Fig. 4a). In contrast, L-[^3^H]serine transport by both Glt_Ph_-R397C and Glt_Ph_-G396S/R397C is inhibited by several ASCT2 selective substrates (Fig. 4b, c). The most notable inhibition is observed for L-cysteine, L-methionine and L-valine, which reduce Glt_Ph_-R397C-mediated L-[^3^H]serine transport by 93 ± 1%, 70 ± 3% and 84 ± 3%, respectively, and reduce Glt_Ph_-R397C/G396S-mediated L-[^3^H]serine transport to a similar extent (Fig. 4b, c; *n* ≥ 3). These results demonstrate that the single point mutation, R397C, increases the promiscuity of Glt_Ph_ towards several neutral amino acids, generating an ASCT2 substrate selectivity phenotype.

Most known inhibitors of the SLC1A family are amino-acid derivatives^19^, therefore it was hypothesised that mutating R397 and G396 in Glt_Ph_ would also affect the inhibitor selectivity profile. The aspartate-based inhibitor L-threo-β-oxybenzylaspartic acid (TBOA) is a known potent inhibitor of Glt_Ph_ and the EAATs^39,48–50^. In wild-type Glt_Ph_, TBOA inhibits L-[^3^H]aspartate transport with an IC_50_ of 2 ± 1 µM (Fig. 4d; *n* ≥ 3), whereas the neutral amino-acid derivatives GPNA, benzylserine and benzylcysteine are ineffective (Fig. 4d; Supplementary Figure 1). L-[^3^H]serine transport mediated by Glt_Ph_-R397C is poorly inhibited by TBOA and GPNA however, benzylserine is an effective inhibitor with an IC_50_ of 110 ± 10 µM (Fig. 4e; *n* ≥ 3). Benzylcysteine also inhibits Glt_Ph_-R397C mediated L-[^3^H]serine uptake, albeit with reduced affinity compared with benzylserine (Fig. 4e). The double mutant transporter, Glt_Ph_-G396S/R397C displays increased inhibition by TBOA compared with Glt_Ph_-R397C, however this inhibition is lower when compared with wild-type Glt_Ph_ (IC_50_ of 500 ± 200 µM compared with 2 ± 1 µM for wild type, Fig. 4f; *n* ≥ 3). GPNA remains a poor inhibitor of Glt_Ph_-G396S/R397C, whereas benzylserine and benzylcysteine are effective inhibitors with IC_50_’s of 120 ± 20 μM and 280 ± 60 μM, respectively (Fig. 4f; *n* ≥ 3).

### Structural basis for substrate selectivity

**Fig. 5:**
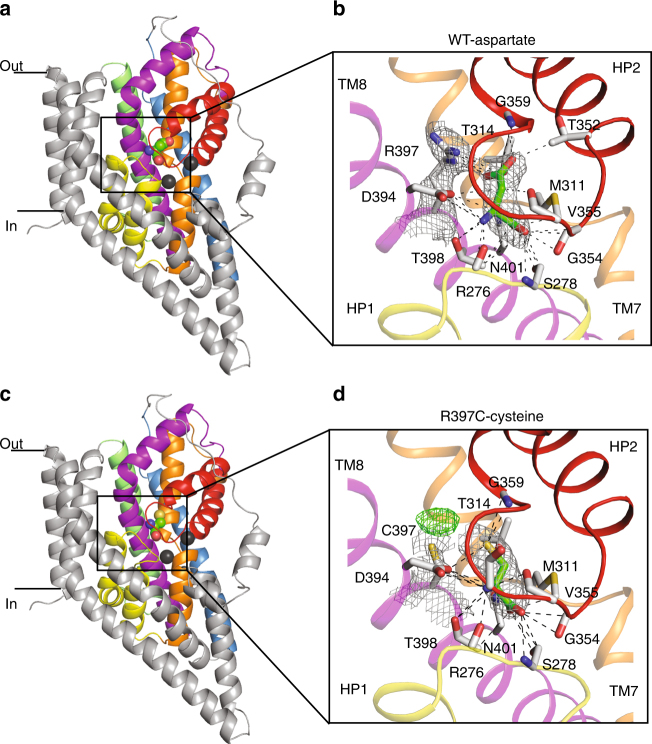
Substrate-binding site in wild-type and mutant Glt_Ph_ View of a single protomer of the wild-type Glt_Ph_ bound to L-Aspartate **a** and Glt_Ph_-R397C bound to L-Cysteine **c**. The protomer is divided into the ‘scaffold domain’ (TM1, TM2, TM4 and TM5, coloured in grey) and the ‘transport domain’ (TM3 in blue, TM6 in green, TM7 in orange, TM8 in magenta, HP1 in yellow and HP2 in red). Bound substrate is shown in stick representation and Na1 and Na2 are shown as dark grey spheres. Close-up view of the L-Aspartate **b** and the L-Cysteine **d** binding sites, respectively. Interactions within 3.5 Å between bound substrate and residues in the Glt_Ph_-binding site are shown as dashed lines. Averaged *2fo–fc* electron density maps at 1σ are shown in dark grey mesh and averaged *fo–fc* electron density maps at 3σ sigma are shown in green

To investigate the structural basis for the altered substrate selectivity profile of Glt_Ph_-R397C, we crystallised this single cysteine mutant transporter in the presence of L-serine, L-cysteine and benzylcysteine (Table 2). In contrast to previously published structures of Glt_Ph_ that utilise a seven histidine mutant known as CAT-7^28^, we used wild-type Glt_Ph_ as a background to introduce mutations. The structure of wild-type Glt_Ph_ in the presence of aspartate was solved and is similar to previously reported CAT-7 structures although the 3-4 loop, which is often disordered is now observed and there are an additional three residues in the N-terminus that are also resolved (Fig. 5a, b; Supplementary Figure 3a, 4a and Table 2). The single mutant transporter Glt_Ph_-R397C was purified in the presence of 5 mM L-serine or L-cysteine and crystallised under the same conditions as wild-type Glt_Ph_^28^. These structures are overall similar to those of L-aspartate bound Glt_Ph_ and are in the outward-occluded conformation, although there are some interesting differences (Fig. 5c, d and Supplementary Figure 3b, 4b). In the L-cysteine-bound Glt_Ph_-R397C structure, the side chain of the cysteine at position 397 is pointing away from the substrate-binding site, which results in an increase in the size of the available pocket (Fig. 5d). Cysteine binds in a similar position to aspartate in wild-type Glt_Ph_ and HP2 is closed, but the space in the binding site created by the removal of Arg397 appears to be occupied by an extra non-protein density (Fig. 5d). We ruled out possibility that bound L-cysteine forms a disulphide bond with Cys397 as the thiols appear to be too far from each other and because similar extra density is observed in crystals of L-serine bound Glt_Ph_-R397C (Supplementary Figure 5a). In the L-serine-bound Glt_Ph_-R397C structure, we observe electron density corresponding to both open and closed conformations of HP2. In one protomer, HP2 was mostly closed; in the other two, we observed strong *fo–fc* density corresponding to the open HP2 conformation (Supplementary Figure 5a). Because of the moderate resolution of the data, we have refrained from explicitly modelling alternate conformations of HP2 and refining their occupancies. It is not clear why there are differences between protomers in the extent of HP2 opening, as crystal packing, which is similar in all our crystals, does not seem to restrict motions of HP2 in either of the subunits (Supplementary Figure 5). Electron density for bound L-serine and accompanying extra non-protein density were well resolved in sharpened threefold averaged maps, but it was not clear whether serine is present at full occupancy in all protomers.

**Table 2:** Data collection and refinement statistics

	Wt/Asp	R397C/B-Cys	R397C/Cys	R397C/Ser
Data collection				
Space group	P61	P61	P61	P61
Cell dimensions				
* a*, *b*, *c* (Å)	114.73, 114.73, 322.51	114.66, 114.66, 321.45	115.31, 115.31, 322.27	114.75, 114.75, 322.24
*α*, *β*, *γ* (°)	90.00, 90.00, 120.00	90.00, 90.00, 120.00	90.00, 90.00, 120.00	90.00, 90.00, 120.00
Resolution (Å)	49.1–3.4	49.07–3.70	49.34–3.80	47.48–3.90
* R*_sym_ or *R*_merge_	7.6 (77.0)	7.8 (55.2)	10.3 (74.1)	6.3 (77.8)
* I*/*σ* *I*	5.8 (0.9)	8.7 (1.7)	6.2 (1.4)	8.5 (1.9)
Completeness (%)	97.2 (99.1)	97.5 (98.9)	96.9 (98.4)	94.9 (95.8)
Redundancy	3.0 (2.9)	3.5 (3.4)	3.0 (3.0)	3.1 (3.0)
Refinement				
Resolution (Å)	39–3.4	39–3.7	39–3.8	20–3.9
No. reflections	31,934	24,729	23,038	20,569
* R*_work_/*R*_free_	24.1/28.7	24.4/24.6	26.2/29.1	22.58/24.65
No. atoms	9051	9030	9030	8893
Protein	9045	9027	9024	8889
Ligand/ion	3	3	3	3
* B*-factors	140.93	185.61	160.93	182.31
Protein	140.95	185.62	160.97	182.33
Ligand/ion	106.27	161.40	99.52	133.23
R.m.s. deviations				
Bond lengths (Å)	0.008	0.007	0.013	0.008
Bond angles (°)	1.07	1.00	1.08	1.04
Ramachandran (%)				
Favoured	94.91	94.76	95.3	96.97
Allowed	4.84	4.18	4.2	2.69
Disallowed	0.25	1.06	0.5	0.34

The structures described above demonstrate that binding of small amino acids, such as L-cysteine and L-serine, to Glt_Ph_-R397C, permits the closure of HP2 as is observed for wild-type Glt_Ph_ in the presence of its substrate L-aspartate (Fig. 5)^39^. This suggests that in addition to L-serine and L-alanine, L-cysteine should also act as a substrate for Glt_Ph_-R397C. To investigate this, we examined L-[^35^S]cysteine uptake into liposomes containing Glt_Ph_-R397C and observe similar levels of uptake of L-cysteine in the presence and absence of the reducing agent dithiothreitol (Supplementary Figure 6a). We also observe robust L-[^35^S]cysteine uptake for ASCT1 and ASCT2 expressed in oocytes (Supplementary Figure 6b), confirming that L-cysteine is a transportable substrate of the ASCTs. None of the other ASCT2 substrate amino acids that inhibit serine transport via Glt_Ph_-R397C (see Fig. 4b) are able to be transported (Supplementary Figure 6c).

**Fig. 6:**
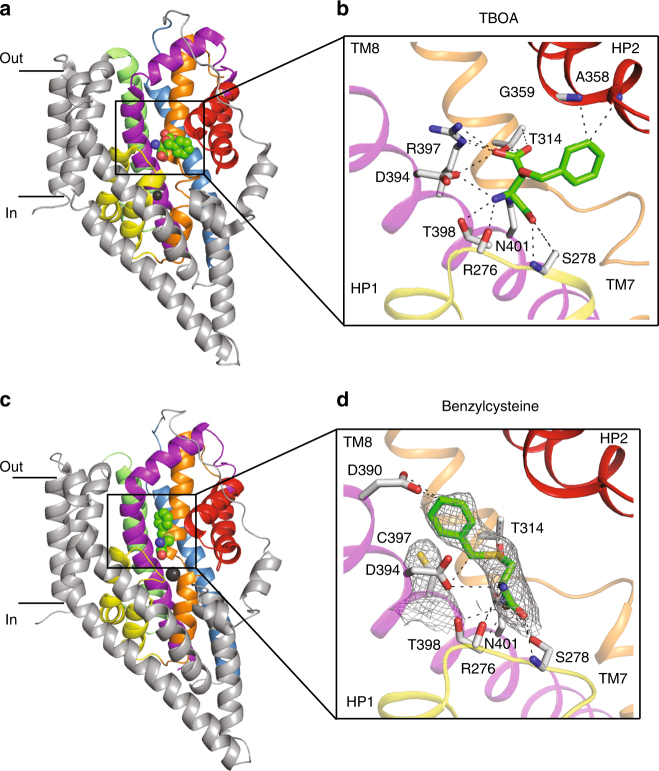
Inhibitor-binding site in wild-type and mutant Glt_Ph_. View of a single protomer of the wild-type Glt_Ph_ bound to TBOA (PDB:2NWW, **a** and Glt_Ph_-R397C bound to benzylcysteine **c**. The protomer is divided into the ‘scaffold domain’ (TM1, TM2, TM4 and TM5, coloured in grey) and the ‘transport domain’ (TM3 in blue, TM6 in green, TM7 in orange, TM8 in magenta, HP1 in yellow and HP2 in red). Bound inhibitor is shown in stick representation and Na1 and Na2 are shown as dark grey spheres. Close-up view of the TBOA **b** and benzylcysteine **d** binding sites. Interactions within 3.5 Å between bound inhibitor and residues in the Glt_Ph_ binding site are shown as dashed lines. Averaged *2fo–fc* electron density maps at 1σ in dark grey mesh are shown for benzylcysteine and C397

The structure of Glt_Ph_-R397C in the presence of benzylcysteine reveals that this inhibitor binds to the substrate-binding site, with HP2 in an open conformation, similar to that observed in the Glt_Ph_-TBOA structure (Fig. 6a, d; Supplementary Figure 3c, 4d). The cysteine moiety of benzylcysteine binds in a similar position to L-cysteine, and the benzyl ring attached to the sulphur occupies the space vacated by arginine in R397C mutant where it may make an additional contact with D390 (TM8) (Fig. 6d). Unlike TBOA, benzylcysteine does not appear to make direct contact with HP2 and it is not immediately clear why the loop is unable to close. L-Glutamine is not a substrate of Glt_Ph_-R397C but is able to bind and inhibit this mutant transporter (Fig. 4b), suggesting that it does not allow full closure of HP2.

To determine whether HP2 prefers an open conformation when the transporter is bound to the inhibitors benzylcysteine and L-glutamine, we used a fluorescence-based assay (see Supplementary methods) to investigate the change in Na^+^-coupling due to the expected loss of the Na2 site, which is formed between HP2 and TM7 when HP2 is closed^39,51^. In Glt_Ph_-R397C, L-serine and L-cysteine binding is associated with an apparent number of coupled Na^+^ ions ~ 1.5–1.8 (Supplementary Figure 7, 8 and Supplementary Table 1), whereas binding of L-aspartate to the wild-type Glt_Ph_ appears to be coupled to ~ 2.9 Na^+^ ions^51^. Thus, binding of aspartate and the expected three Na^+^ ions is an almost fully cooperative process in the wild-type transporter. The decreased number of coupled ions in Glt_Ph_-R397C is either due to the loss of a Na^+^-binding site or to the diminished cooperativity of binding. The latter possibility seems more likely because the geometry of all three Na^+^ sites is preserved, at least in cysteine-bound Glt_Ph_-R397C, and it is unlikely that some of these sites are unoccupied. In addition, reduced substrate and Na^+^ ion coupling was previously observed when Arg397 was replaced with the unnatural amino-acid citrulline lacking positive charge^52^. An apparent number of ions coupled to binding of L-glutamine and benzylcysteine is further reduced to ~ 1 as would be expected if Na2 site were not used. These results are consistent with previous work demonstrating reduced Na^+^ coupling of TBOA binding compared with aspartate in wild-type Glt_Ph_^51^.

## Discussion

A proposed cancer treatment is to starve cancer cells of glutamine using ASCT2 inhibitors, thereby impairing growth and proliferation^8^. To develop subtype selective, high-affinity inhibitors of ASCT2 information regarding the binding of neutral amino acids and the molecular basis for the differences in substrate selectivity between ASCT1 and ASCT2 is required. In this study, we use site-directed mutagenesis coupled with functional and structural characterisation to investigate the structural features that define the binding site of the ASCTs. Insights provided through understanding the molecular determinants for substrate specificity in ASCT1 and ASCT2 may be utilised in drug design to selectively target ASCT2.

One of the main substrate interactions in the EAATs and Glt_Ph_ is between R397 in TM8 and the side chain carboxylate group of the bound aspartate/glutamate (Fig. 1b). This residue is critical in determining acidic versus neutral amino-acid selectivity in the SLC1A family^42,44,45^. Here, we have shown that the equivalent residues in ASCT1 and ASCT2 (threonine and cysteine, respectively) are also responsible for determining glutamine selectivity (Fig. 2). This is the first demonstration of how ASCT1 and ASCT2 establish two distinct substrate selectivity profiles. Although the single T459C mutation in ASCT1 was enough to introduce glutamine transport, an additional mutation outside of the binding site was required to create a binding site more similar to WT-ASCT2 and also to introduce inhibitor GPNA binding to ASCT1. This additional mutant, T458S is unlikely to play a direct role in coordination of substrate/inhibitor in the binding site but may alter the positioning of TM8 and thus the size/chemistry of the binding pocket and is reminiscent of other ‘second shell’ mutations that play a role in substrate selectivity of a bacterial homologue of the SLC6 family^53^. Mutation of the equivalent arginine in Glt_Ph_ to the ASCT2 counterpart (R397C) switches the substrate selectivity from acidic to neutral amino acids (Fig. 3), in agreement with similar studies performed in the EAATs^42–44^ and ASCT1^45^. Remarkably, R397C in Glt_Ph_ also increased the binding of several neutral amino acids that are substrates of ASCT1/2 with serine, alanine and cysteine becoming transportable substrates of Glt_Ph_-R397C (Fig. 4 and Supplementary Figure 6). This broadened substrate selectivity may be due to the increased size of the binding site due to the removal of the bulky, positively charged side chain from R397. The addition of a second mutation equivalent to T458 in Glt_Ph_ did not further alter substrate/inhibitor selectivity, which could be a result of sequence differences in the region between TM8 and TM6 in Glt_Ph_ and the ASCTs.

The structures of Glt_Ph_-R397C reveal a very similar conformation to the outward-occluded structure of wild-type Glt_Ph_^39^, in which HP2 is closed down over the bound L-serine or L-cysteine with Na2 also likely bound. L-cysteine is bound in all three protomers, and although density is observed for L-serine it is not clear whether it is present at full occupancy in all protomers. This may be due to the relatively low affinity of serine for Glt_Ph_-R397C compared with that of L-aspartate for wild-type Glt_Ph_ (100-fold lower affinity). In both the L-cysteine- and L-serine-bound structures, the thiol moiety of the cysteine residue introduced at position 397 is pointing away from the binding site creating a space vacated by the arginine side chain that contains a non-protein density. We have attempted to model water or Na^+^ into this density, but at the current resolution, we are not confident in its assignment. Nonetheless, serine, alanine and cysteine are clearly transported substrates of Glt_Ph_-R397C (Fig. 3 and Supplementary Figure 6).

In this study, we have demonstrated for the first time that GPNA is selective for ASCT2 over ASCT1. Surprisingly, the introduction of L-glutamine transport in ASCT1-T359C, and L-glutamine binding in Glt_Ph_-R397C is not sufficient to introduce inhibition by the glutamine-derivative GPNA. However, the additional T458S mutation in ASCT1 does increase the apparent affinity of L-glutamine and GPNA to more closely resemble that of ASCT2 (Fig. 2). The side chain of T458 points away from the substrate-binding pocket towards the adjacent helix of TM6, where it is likely to interact with residues Y236 and L239 (Fig. 1c). We hypothesise that mutation of T458 alters interactions between TM8 and TM6, thereby affecting the position of TM8 and altering the conformation of the substrate-binding pocket to allow binding of the larger amino-acid L-glutamine. We propose that T459C is required for the recognition of L-glutamine-based compounds in ASCT1 and a change in binding site conformation, such as that induced by T458S, is required to fit larger compounds.

In Glt_Ph_, the double mutant G396S/R397C displays an increased apparent affinity for L-alanine, however the transporter remains inefficient at binding L-glutamine and glutamine-based inhibitors. Despite this, the single and double mutant Glt_Ph_ transporters display an altered inhibitor selectivity profile. Benzylserine and benzylcysteine become effective inhibitors, whereas the TBOA can no longer bind. Like TBOA, benzylserine and benzylcysteine are composed of an amino-acid backbone with an additional benzyl ring, although the benzyl ring is attached to the side chains of serine and cysteine rather than to the β carbon as in TBOA. The structure of Glt_Ph_-R397C in the presence of benzylcysteine suggests that these inhibitors have a similar mechanism of action to TBOA on the glutamate transporters, where the amino-acid moiety fits into the substrate-binding site, and the benzyl ring prevents the closure of HP2 and entry into a transport-competent state. In TBOA-bound wild-type Glt_Ph_, the benzyl group on the β carbon props HP2 open (Fig. 6a, b). By contrast, in the benzylcysteine-bound Glt_Ph_-R397C, the benzyl group is an extension of the side chain and occupies the space created by the R397C mutation (Fig. 6c, d). It does not make any direct contacts with HP2. This result suggests that if HP2 cannot close down over the binding site, it preferentially remains in an open state and interacts with the 3–4 loop as is observed in complex of wild-type Glt_Ph_ with TBOA. Our data may also explain the relatively low affinity of benzylserine and benzylcysteine for the ASCTs (~ 1 mM) compared with the affinity of TBOA for the glutamate transporters, which makes more extensive contacts with residues in HP2 (~ 50 μM)^48^.

Analysis of the substrate-binding pocket in the L-cysteine-bound and benzylcysteine-bound Glt_Ph_-R397C structures reveals that the pocket is too small to accommodate benzylcysteine. Significant clashes were observed between benzylcysteine and residues in HP2 when the loop is closed, explaining why this compound serves as a blocker (Supplementary Figure 9). In contrast, there are no observed clashes between cysteine and closed HP2, supporting the finding that cysteine is a substrate of Glt_Ph_-R397C as HP2 is required to close down over the binding site for translocation to occur. At the current resolution of these structures, it appears that all compounds bind in a similar pose, suggesting that coordination of the main chain atoms of the compounds by residues in HP1 and TM8 are crucial for binding. In contrast, interactions with HP2 that are observed in the L-aspartate and L-cysteine-bound structures may be less important, perhaps explaining why inefficient closure of HP2 is observed in the L-serine bound structure, even though no clashes between L-serine and HP2 are expected. Here, minor differences in how the compounds bind to the protein may lead to a less favourable closed conformation of HP2 and thus determine whether a compound acts as a substrate or a blocker.

The reduced Na^+^ coupling of the inhibitors (benzylcysteine and L-glutamine) compared with substrates (cysteine and serine) in Glt_Ph_-R397C supports the structural data showing that HP2 is open in the inhibitor-bound structure. If HP2 is unable to close down over the substrate-binding site, the binding site for the second Na^+^ ion (Na2) cannot be not formed^39^. However, the reasons for the reduced Na^+^-coupling of L-serine and L-cysteine in Glt_Ph_-R397C compared with that of L-aspartate in wild-type Glt_Ph_ is less obvious. A recent study of a closely related transporter, Glt_Tk_, revealed that Na^+^-binding induces a twisting of a highly conserved NMDGT motif in TM7, such that threonine at position T314 displaces the substrate-coordinating R397, opening up the substrate-binding pocket to allow aspartate to bind^20^. In Glt_Ph_-R397C, the small side chain of cysteine is less likely to undergo such large conformational changes, and therefore T314, and by extension Na^+^ binding, will have a lesser effect on the conformation of the substrate-binding pocket. We have also demonstrated that although Na^+^ is likely to bind at all three Na^+^ sites in ASCT1, binding at all three sites is not necessary for transport^32^. The proposed reduced effects of Na^+^-induced twisting of the NMDGT motif may explain the reduced Na^+^-coupling in the ASCTs and Glt_Ph_-R397C compared with transporters that have an arginine residue at this position. Indeed tighter Na^+^ binding and weaker coupling between substrate and Na^+^ binding has also been observed in a Glt_Ph_ variant where R397 was replaced by a neutral citrulline moiety using chemical protein ligation^52^.

Interestingly, the transport of neutral amino acids by Glt_Ph_-R397C remained concentrative, unlike transport via the EAATs, where a similar mutation renders the transporter an obligate amino-acid exchanger^44^. In the EAATs, the equivalent mutation impaired K^+^ interactions, and it was proposed that this was the cause of inducing obligatory exchange mechanism, in a similar fashion to other mutant EAATs that had impaired K^+^ interactions^54,55^. The lack of K^+^ coupling in Glt_Ph_ may explain why it does not share the same shift to exchange mode as the EAATs when transporting neutral amino acids. However, the link between substrate/ion translocation and transport versus exchange mechanisms is poorly understood and warrants further investigation.

**Fig. 7:**
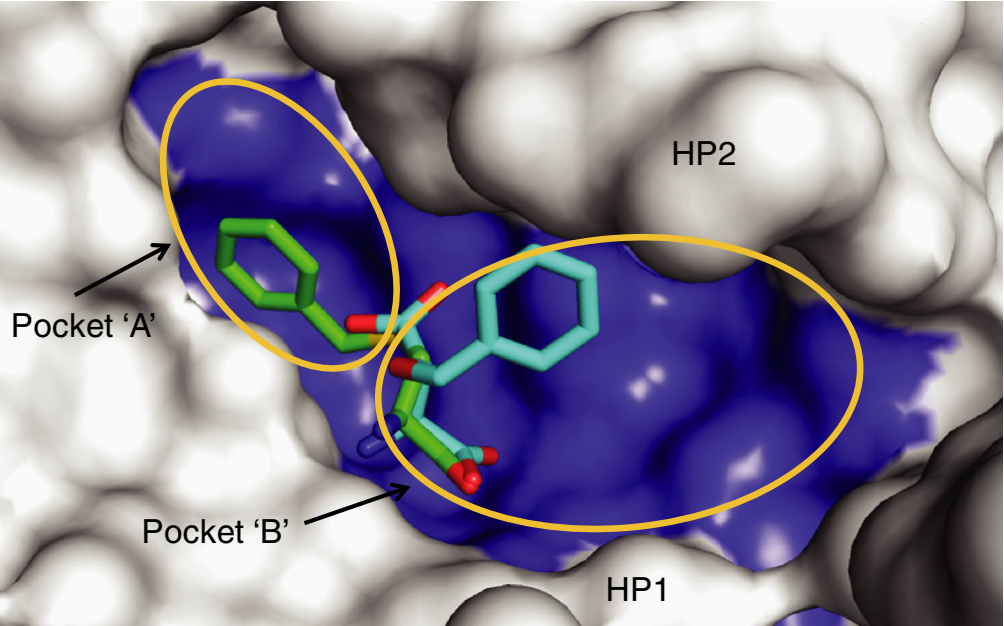
Binding pocket of Glt_Ph_-R397C Surface representation of the binding pocket of Glt_Ph_-R397C bound to benzylcysteine (green sticks). TBOA bound to Glt_Ph_ (PDB:2NWW; cyan sticks) is superimposed in the binding site. ‘Pocket A’ and ‘Pocket B’ indicated by orange circles

In this study, we have identified two residues in ASCT1/2 that are involved in determining differences in their substrate and inhibitor selectivity profiles. A subtle change in the properties of the binding site was shown to play an important role in selectivity, as influenced by residue interactions outside of the binding site. In the absence of a structure of human ASCT2, Glt_Ph_-R397C provides a model of the ASCT2 substrate-binding site, which may facilitate drug design. Our functional and structural data suggest adding bulk off the side chain of the neutral amino acids (pocket A) in addition to the β carbon (pocket B) may generate compounds that make more extensive contacts with the open-to-out binding site of ASCT2 (Fig. 7), in agreement with docking studies on an ASCT2 homology model^56,57^. These findings may inform the development of selective, higher affinity ASCT2 inhibitors to treat glutamine-dependent cancers.

## Methods

### Site-directed mutagenesis

ASCT1 and ASCT2 were subcloned into the plasmid oocyte transcription vector, and Glt_Ph_ was subcloned into the bacterial expression vector pBAD. Site-directed mutagenesis was performed using the Q5 Site-Directed Mutagenesis Kit (New England BioLabs Inc.). Primers were designed using NEBaseChanger (New England BioLabs Inc.) and synthesised by Sigma Genosys (Sydney, Australia). Primer sequences are listed in Supplementary Table 2. DNA sequences of all mutations were confirmed by the Australian Genome Research Facility (Sydney, Australia). DNA was prepared using the PureLink Quick Plasmid Miniprep Kit (Life Technologies), cDNA was linearised with Spe1 (Promega) and mRNA transcribed with T7 polymerase using the mMESSAGE mMACHINE kit (Ambion).

### Electrophysiology

All chemicals were obtained from Sigma unless otherwise stated. Stage V oocytes were harvested from female *Xenopus laevis* (frogs) which were obtained from Nasco (Fort Alkinson, USA). Frogs were anaesthetised with 6.5 mM tricaine neutralised with 7.14 mM sodium bicarbonate. After making an incision in the abdomen of the frog, ovarian sacs were removed and stored in OR-2 buffer (82.5 mM NaCl, 2 mM KCl, 1 mM MgCl_2_, 5 mM hemisodium HEPES, pH 7.5). This surgical procedure follows a protocol (#2016/970) approved by the University of Sydney Animal Ethics Committee under the Australian Code of Practice for the Care and Use of Animals for Scientific Purposes. To isolate oocytes, the ovarian sacs were cut into small sections before being digested by agitation with 2 mg per ml Collagenase A for 1–2 h. Following digestion, defoliculated oocytes were initially rinsed with OR-2, followed by frog Ringer’s solution (96 mM NaCl, 2 mM KCl, 1 mM MgCl_2_, 1.8 mM CaCl_2_, 5 mM hemisodium HEPES, pH 7.5). A total of 20 ng of transporter cRNA was injected into oocytes which were stored in frog Ringer’s solution supplemented with 0.1% gentamycin, 50 μg per ml tetracycline, 2.5 mM pyruvate and 0.5 mM theophylline at 16–18 °C.

After 2–4 days of microinjection, current recordings were made using the two electrode voltage clamp technique with a Geneclamp 500 amplifier (Axon Instruments, Foster City, CA) interfaced with a MacLab 2e chart recorder (ADI Instruments, Sydney, Australia) using the chart software, and a Digidata 1322A (Axon Instruments) controlled by an IBM-compatible computer using the pClamp software (version 10, Molecular Devices, Union City, CA). The current–voltage relationships for substrate elicited conductances were obtained by subjecting cells to 200 ms voltage pulses between −100 mV and + 60 mV in 10 mV steps. Current–voltage relationships were calculated by subtracting steady state current measurements in the absence of substrate from corresponding current measurements in the presence of substrate.

Recording solution for all experiments (except where otherwise stated) was normal frog Ringer’s solution (Cl^−^-containing buffer) with complete NO_3_^−^ substitution for Cl^−^. For Na^+^ titrations, NMDG^+^ was used as the substitute cation. The pH of recording solutions was adjusted using HNO_3_ and NaOH or KOH. Recordings were made with the bath grounded via a 3 M KCl/agar bridge connected to a 3 M KCl reservoir to minimise offset potentials. Cells were washed with Cl^−^-containing buffer between substrate applications to ensure that NO_3_ loading of the cell was not significant. Current (*I*) as a function of substrate concentration was fitted by least-squares analysis to a derivation of the Michaelis-Menten equation, *I = I*_max_ • [substrate]/([substrate] + EC_50_), where *I*_max_ is the maximum current generated and EC_50_ is the substrate concentration which generates a half-maximal response. Na^+^ concentration responses were fit to the Hill equation, *I*/*I*_max_ = [substrate]^*n*^/([substrate]^*n* + ^(EC_50_)^*n*^) where *n* is the Hill coefficient and all other terms are as described above. All data presented are from *n* > 3 from at least three separate batches of oocytes.

### Radiolabelled uptake in oocytes

Uptake of L-[^3^H]amino acids (PerkinElmer Life Sciences) was measured in oocytes expressing wild-type and mutant ASCT1, and uninjected oocytes. Five oocytes were incubated in Cl^−^-containing buffer with 10 µM L-[^3^H]amino acids at room temperature. After 10 min, uptake was terminated by three rapid washes in ice-cold Cl^−^-containing buffer followed by lysis in 50 mM NaOH and 1% SDS. L-[^3^H]amino acids were measured by scintillation counting using a Trilux beta counter (PerkinElmer Life Sciences).

### Protein purification and reconstitution

Mutant and wild-type Glt_Ph_ with an amino-terminal hexa-His tag were expressed in *Escherichia coli* Top10 cells (Invitrogen), which were grown in Luria broth medium at 37 °C. Ampicillin was added to a final concentration of 100 μg per ml. To induce expression of Glt_Ph_, 0.1% L-arabinose was added when cells reached an optical density of 0.6 at 660 nm, and cells were harvested 4 h post induction. Membranes were isolated, and then solubilised with n-dodecyl-β-D-maltopyranoside (C_12_M, Anatrace). Histidine containing proteins were purified using Ni^2+−^nitrilotriacetic acid resin (Qiagen). The histidine tag was subsequently removed from Glt_Ph_ by digestion with thrombin (10 U per mg protein) and the protein further purified on a Superdex 200 size exclusion column, where the detergent was exchanged to n-decyl-β-D-maltopyranoside (C_10_M, Anatrace).

Pure protein was reconstituted into liposomes composed of 75% *E. coli* Polar Lipid Extract and 25% 1-palmitoyl-2-oleoyl-*sn*-glycero-_3_^−^phopshocholine (Avanti Polar Lipids). Lipids were mixed, dried under nitrogen and resuspended in internal buffer (100 mM KCl, 20 mM HEPES-Tris pH 7.5) using brief sonication (Laboratory Supplies Co.). The lipid suspension was frozen in liquid nitrogen and thawed at room temperature several times. Liposomes were formed by extrusion through 400 nm polycarbonate membranes (Avanti Polar Lipids) and were treated with Triton X-100 at a 0.5:1 (w/w) detergent to lipid ratio prior to the addition of protein at 0.25 μg protein per mg lipid^37^. The protein/lipid mixture was left at room temperature for 30 min before detergent was removed using SM2 Biobeads (BioRad). The mixture was then incubated with gentle agitation, with four consecutive batches of Biobeads (80 mg per ml). Proteoliposomes were concentrated by centrifugation at 150,000 *g* for 30 min, resuspended at 100 mg lipid per ml and either used immediately or flash-frozen in liquid nitrogen and stored at −80 °C.

### Proteoliposome transport assay

Mutant and wild-type Glt_Ph_-mediated transport of radiolabelled substrate was assayed by diluting proteoliposomes (100 mg lipid per ml) 133-fold into uptake buffer (100 mM NaCl, 20 mM HEPES-Tris pH 7.5, 1 μM valinomycin, 100 nM–1 µM L-[^3^H]substrate) pre-warmed to 30 °C. At each time point, a 200 µl aliquot was removed and diluted 10-fold into ice-cold quench buffer (100 mM LiCl, 20 mM HEPES-Tris pH 7.5), followed by immediate filtration over nitrocellulose filters (0.22 μm pore size, Millipore). The filters were washed with ice-cold quench buffer and assayed for radioactivity using a Trilux beta counter (PerkinElmer). To establish substrate concentration–response curves, extraliposomal L-[^3^H]substrate was varied between 500 nM and 100 µM, maintaining NaCl concentrations at 300 mM. For inhibitor concentration–response curves, for wild-type Glt_Ph_ 100 nM L-[^3^H]aspartate, and for mutant Glt_Ph_ 1 µM L-[^3^H]serine, was co-applied with varying inhibitor concentrations. Background levels of uptake were measured by diluting proteoliposomes into internal buffer (100 mM KCl, 20 mM HEPES-Tris pH 7.5) containing 1 µM of valinomycin and the indicated concentrations of L-[^3^H]substrate.

### Fluorescence-based binding assays

Fluorescence-based binding assays were performed by titrating purified protein (50 μg per ml) in 20 mM HEPES/Tris, pH 7.4, 200 mM choline chloride, 0.4 mM *n*-dodecyl^−^β-D-maltopyranoside, 200 nM styryl fluorescent dye RH421 (Invitrogen, Inc., Grand Island, NY) with NaCl, L-ser, L-cys or benzylcysteine at 25 °C^51^. The RH421 dye was excited at 532 nm, and the fluorescence was collected at 628 nm using a QuantaMaster (Photon International Technology, Inc., Edison, NJ). Fluorescence emissions were measured after at least 10 min equilibration to ensure a stabilised signal. The data were analysed using GraphPad Prism version 6.0 for Mac (GraphPad Software, La Jolla California USA). Fractional fluorescence changes were normalised to maximal fluorescence changes, then plotted as a function of ligand concentration and fitted to the Hill equation. All the experiments were performed at least in triplicate.

### Crystallography and structure determination

Wild-type Glt_Ph_ and Glt_Ph_R397C was purified as above except that the SEC buffer was 20 mM Hepes/Tris pH 7.5, 25 mM NaCl, 25 mM KCl and 7 mM n-decyl-a-D-maltppyranoside (Anatrace). Crystals were grown at 4 °C using the hanging-drop vapour diffusion method, by mixing protein (~ 7 mg per ml) and well solution (1:1 vol:vol), 15–21% PEG1000, 0.1 M LiSO_4_, 0.05 M Citric acid and 0.05 M Na_2_HPO_4_. Benzylcysteine, cysteine and serine crystals were obtained in the same crystallisation conditions supplemented with 5 mM Benzylcysteine, L-cysteine and L-serine, respectively. Protein crystals were cryoprotected by soaking in the well solution supplemented with 20 to 30% PEG1000, 5% glycerol and 2 m M n-decyl-a-D-malopyranoside. All diffraction data were collected on the ADSC Quantum 315r detector at the Australian Synchrotron beamline MX2 at a wavelength of 0.97 Å^58^. Data sets were indexed, integrated and scaled using XDS^59^. Initial phases were obtained by molecular replacement with Phaser^60^ using the structure of Glt_Ph_ with bound aspartate (PDB:2NWX) or with bound TBOA (PDB:2NWW) as the search model. The protein model was built manually in Coot^61^ and refined using REFMAC5^62^ with TLS and threefold non-crystallographic symmetry (NCS) restraints^63^. Phases were further improved by rounds of manual rebuilding followed by restrained refinement in REFMAC with tight threefold NCS restraints. Statistical analysis for the crystal diffraction data for benzylcysteine-bound transporter suggested that the data might be twinned. However, refinement of the twin fraction in REFMAC5 yielded <10% of the twin. Therefore, untwined refinement was carried out further. Unit cell parameters, data collection and refinement statistics are presented in Table 1. All structural figures were prepared using PyMOL^64^.

### Data availability

All relevant data are available from the corresponding authors upon reasonable request. The coordinates of the refined models and structure factors have been deposited into the Protein Data Bank (PDB repository: 6BAT for Wild-type Glt_Ph_-aspartate; 6BAU for Glt_Ph_-R397C-cysteine; 6BMI for Glt_Ph_-R397C-serine and 6BAV for Glt_Ph_-R397C-benzylcysteine).

## Electronic supplementary material

Supplementary Information
