# Supplementary material for: Structural characterisation reveals insights into substrate recognition by the glutamine transporter ASCT2/SLC1A5
Source: Nat Commun. 2018 Jan 2;9:38. doi: 10.1038/s41467-017-02444-w (PMC5750217; doi:10.1038/s41467-017-02444-w)
Supplement: Supplementary file 1 — Supplementary Information [file 41467_2017_2444_MOESM1_ESM.pdf]

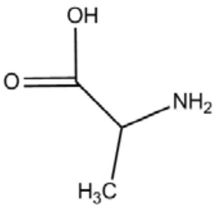

Alanine

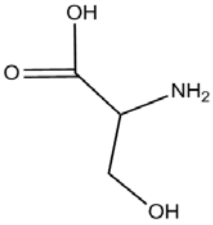

Serine

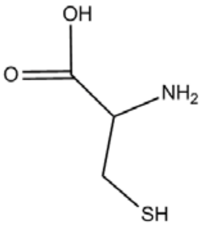

Cysteine

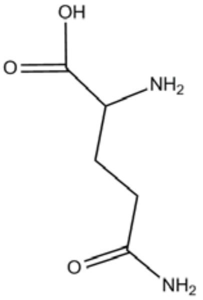

Glutamine

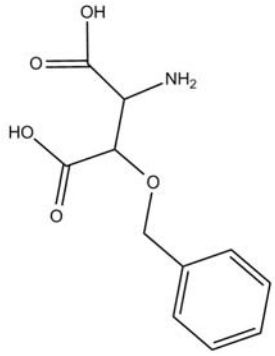

Threo-β-oxybenzylaspartic acid

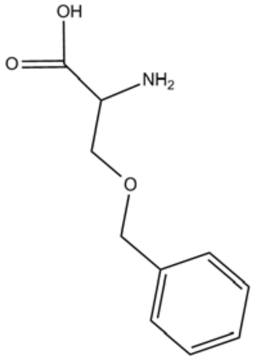

Benzylserine

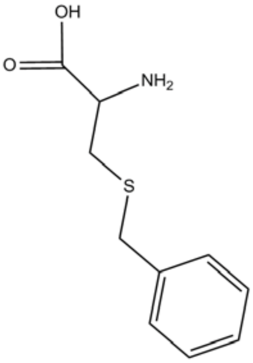

Benzylcysteine

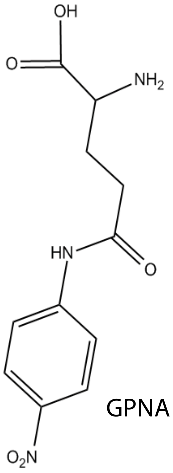

GPNA

Supplementary Figure 1. Chemical structure of substrates/inhibitors of ASCT1/2.

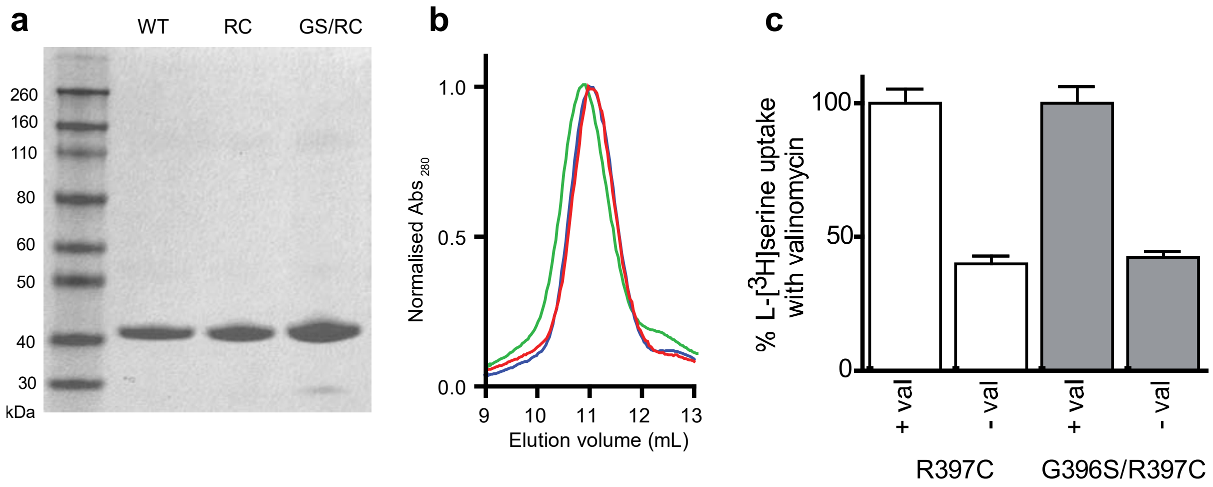

Supplementary Figure 2. Substrate binding site mutations in Glt_{Ph}. (**a**) SDS–PAGE analysis of wild type Glt_{Ph} and mutants Glt_{Ph}-R397C and Glt_{Ph}-G396S/R397C. Detergent-solubilized purified wild type and mutant Glt_{Ph} were visualized by Comassie staining. (**b**) Size exclusion column profile for wild type Glt_{Ph} (red), Glt_{Ph}-R397C (green) and Glt_{Ph}-G396S/R397C (blue; obstructed by red Glt_{Ph} curve). (**c**) Neutral amino acid transport by Glt_{Ph}-R397C and Glt_{Ph}-G396S/R397C is electrogenic. L-[^{3}H]serine transport is shown for both Glt_{Ph}-R397C (white bars) and Glt_{Ph}-G396S/R397C (grey bars) in the presence (+ val) and absence (- val) of valinomycin, as a percentage of L-[^{3}H]serine transport at 30 min in the presence of valinomycin.

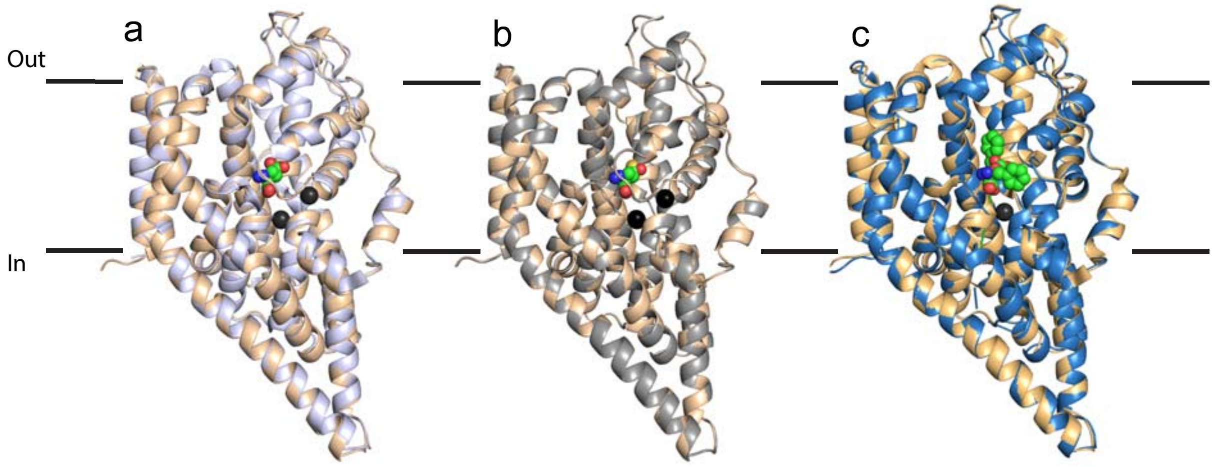

Supplementary Figure 3. Superimposition of the aspartate-bound CAT7_Glt$_{Ph}$ structure in light blue (PDB:2NWX) with the aspartate-bound wild-type Glt$_{Ph}$ structure in light brown (**a**) and with the Glt$_{Ph}$-R397C structure with bound L-cysteine in grey (**b**). Superimposition of the TBOA-bound CAT7_Glt$_{ph}$ structure in gold (PDB:2NWW) with Glt$_{Ph}$-R397C bound to benzylcysteine in blue **(c)**. Substrate/inhibitors are in stick representation and Na1 and Na2 are shown as dark grey spheres.

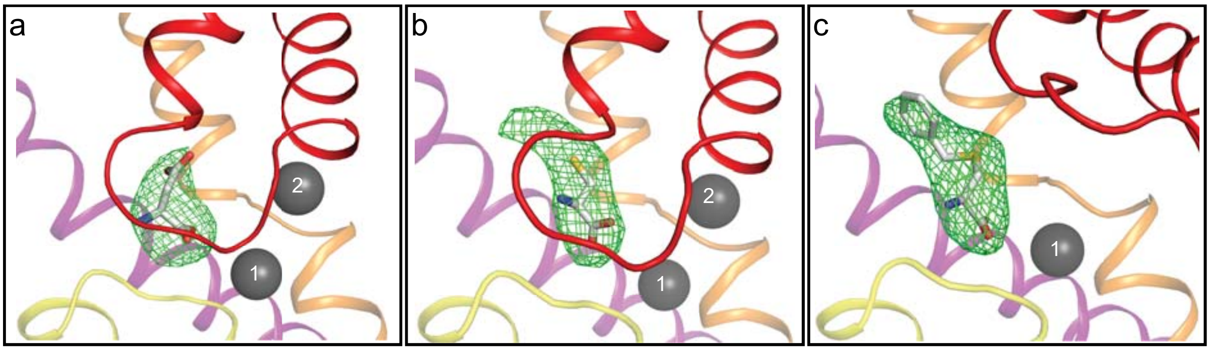

Supplementary Figure 4. **Omit maps of bound substrates/inhibitors**. Wild type Glt_{ph} bound to L-Aspartate (a) and Glt_{Ph}-R397C bound to L-Cysteine (b) and Benzylcysteine (c). Substrate/inhibitor are shown in stick representation; Na1 and Na2 are shown as grey spheres; TM7 (orange), TM8 (magenta), HP1 (yellow) and HP2 (red). Omit maps were generated by removing substrate/inhibitor and applying 0.1 Å random noise to protein coordinates followed by re-refinement of the structures in REFMAC5[1]. Omit maps contoured at 3σ are shown in green and substrate/inhibitor has been placed in the density for reference. We use the position of aspartate bound in Glt_{Ph} (PDB:2NWX) to guide our ligand placement by overlaying the amino acid backbone moiety of the substrates/inhibitors. The placement of aspartate in PDB:2NWX structure was achieved using an alternate compound, L-cysteine sulfinic acid, which shows an anomalous signal from the sulphur atom[2].

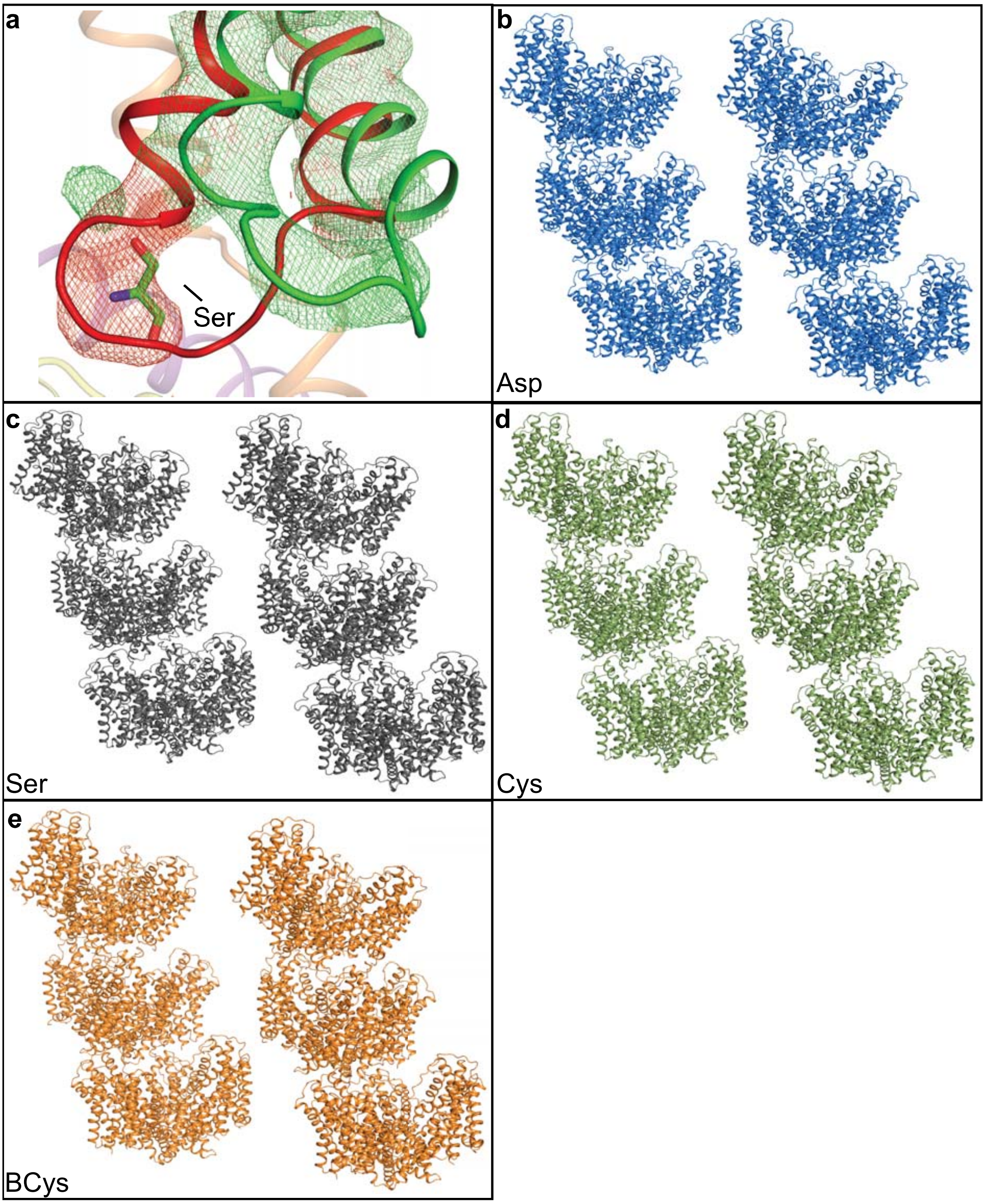

Supplementary Figure 5. HP2 loop configuration for the Glt_{Ph}-R397C bound to L-Serine. (a) Omit maps were generated by removing HP2 and applying 0.1 Å random noise to protein coordinates followed by re-refinement of the structures in REFMAC5[1]. Omit maps contoured at 3σ are shown in green (HP2 open) and red (HP2 closed). HP2 has been placed in the densities for reference. Closed and open conformations correspond to those observed in the crystal structures of Asp- and TBOA-bound wild-type Glt_{Ph} transporter, respectively. The extra non-protein density is also observed in the omit map (green) adjacent to bound serine. Crystal packing of the Glt_{ph} trimers in $P6_1$ for wild type Glt_{Ph} bound to L-Aspartate (b) and R397C-Glt_{Ph} bound to L-Serine (c), L-Cysteine (d) and Benzylcysteine (e).

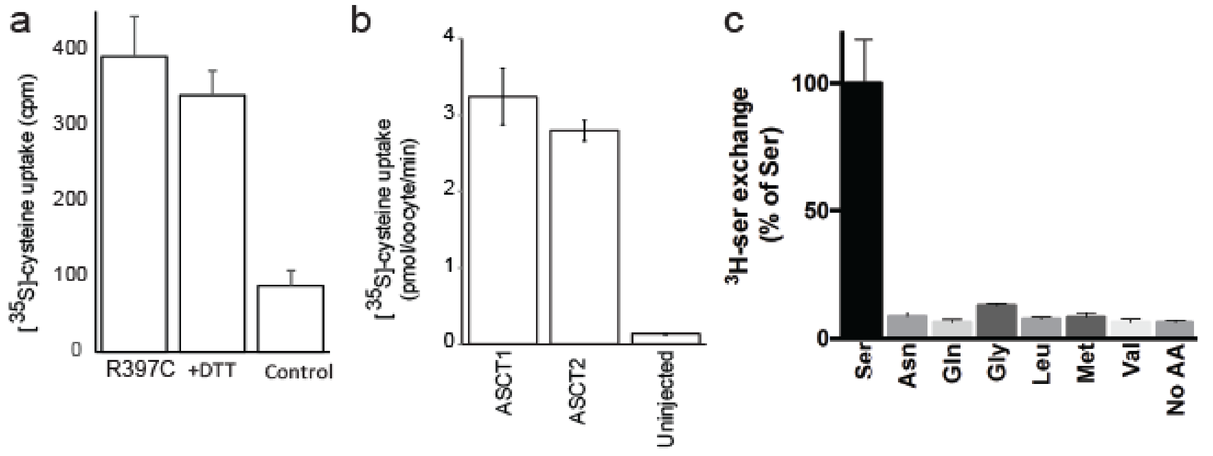

Supplementary Figure 6. Glt_{Ph}-R397C, ASCT1 and ASCT2 are functional cysteine transporters. (**a**) Purified Glt_{Ph}-R397C was reconstituted into liposomes and 10μM ^{35}S-cysteine transport was measured over 10 minutes alone or in the presence of 1 mM dithiothreitol (DTT). Background levels of uptake were measured by diluting proteoliposomes into internal buffer (100 mM KCl, 20 mM HEPES-Tris pH 7.5) containing 1 μM of valinomycin and the 10μM ^{35}S-cysteine. (**b**) Oocytes expressing ASCT1, ASCT2 or uninjected oocytes (control) were incubated in ND96 buffer containing 10 μM ^{35}S-cysteine over 10 mins. (**c**) Neutral amino acid exchange in Glt_{Ph}-R397C. Counterflow experiments in which Na^+ and amino acid loaded liposomes were used to detect the transport of a range of unlabelled neutral amino acids by exchanging with external ^{3}H-serine. Counterflow assays were performed using liposomes containing 200mM NaCl, 20 mM HEPES/Tris pH 7.5, and 1 mM of unlabelled amino acid as indicated. Control liposomes excluded amino acids from the internal buffer (No AA). Loaded liposomes were diluted into buffer containing 200 mM NaCl, 20 mM HEPES/Tris pH 7.5 and 2 μM ^{3}H-serine and incubated at 35°C for 30 min. Data are shown as a percentage of ^{3}H-serine accumulation after 30 min due to exchange with unlabelled internal serine (Ser). All data represent the mean of triplicate experiments ± S.E.M.

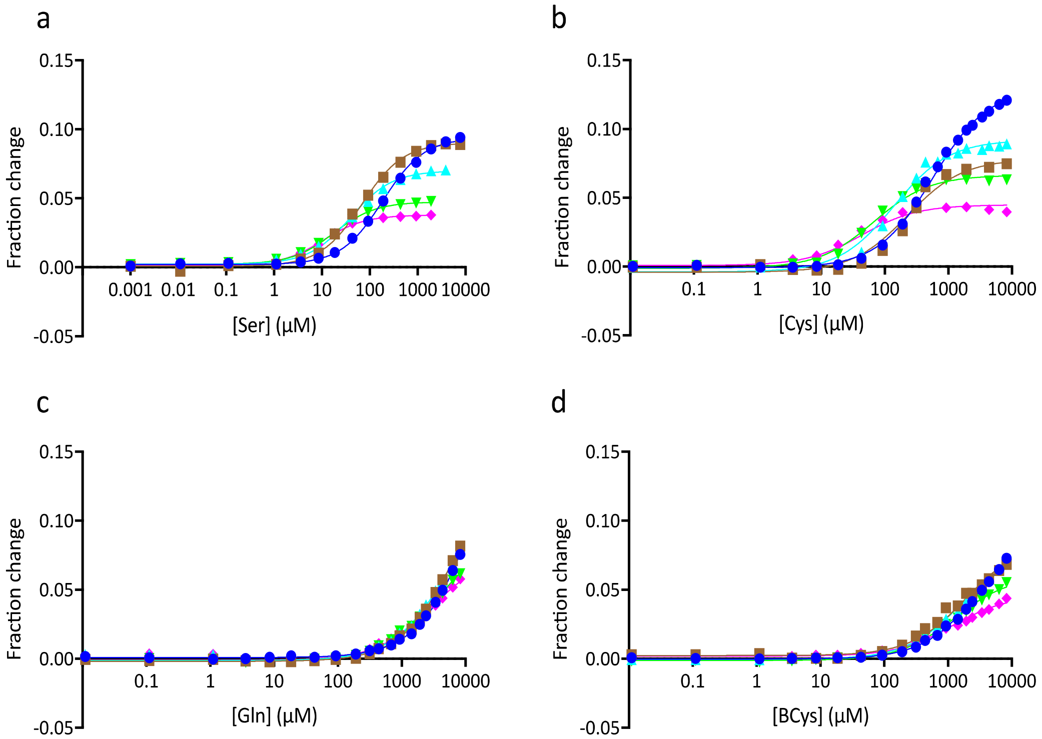

Supplementary Figure 7. Amino acid binding isotherms. Binding isotherms for serine (a), cysteine (b), glutamine (c), and benzyl-cysteine (d) in the presence of Na^+ in mM: 10 (blue), 20 (brown), 30 (cyan), 60 (green), 100 (magenta). Each isotherm is an average of 3-6 measurements. Errors are not shown for clarity. The lines through the data are global fits with *n* values shown in Supplementary Table 1.

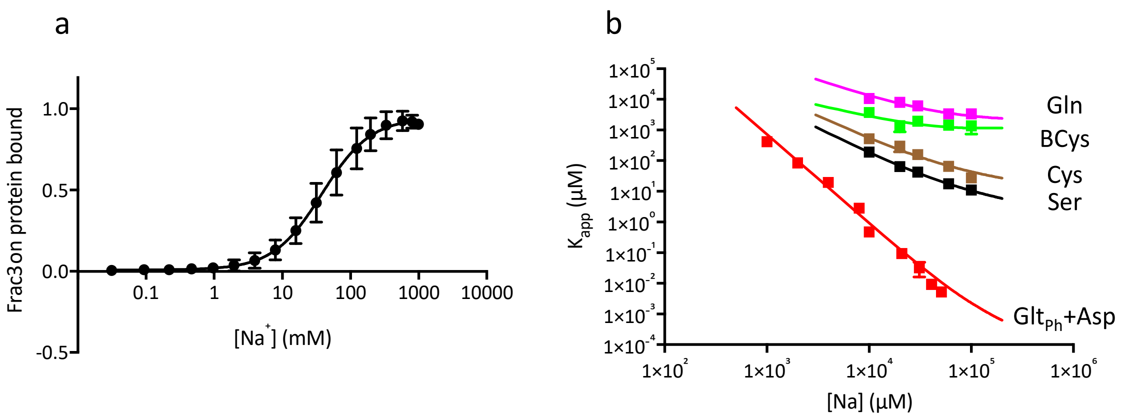

Supplementary Figure 8. Independent Na^+ binding and coupled amino acids and Na^+ binding to Glt_{Ph}-R397C. (a) Na^+ binding isotherm in the absence of amino acids. Data are an average of 6 titrations. The line through the data is a fit to Hill equation with K_D of 38 mM and n_{Hill} of 1.2. (b) Apparent K_D-s of serine (black), cysteine (brown), benzylcysteine (green) and glutamine (purple) binding to Glt_{Ph}-R397C and aspartate binding to wild type Glt_{Ph} protein (red, data reproduced from Reyes et al., 2013[3]). Lines through the data points were simulated using the parameters from the global fits and the simplified binding polynomial. The line through the data for wild type Glt_{Ph} was generated using published values[3]: $K_{D,Na}$ = 99.3 mM, n_{Na} = 1.6, n = 2.9.

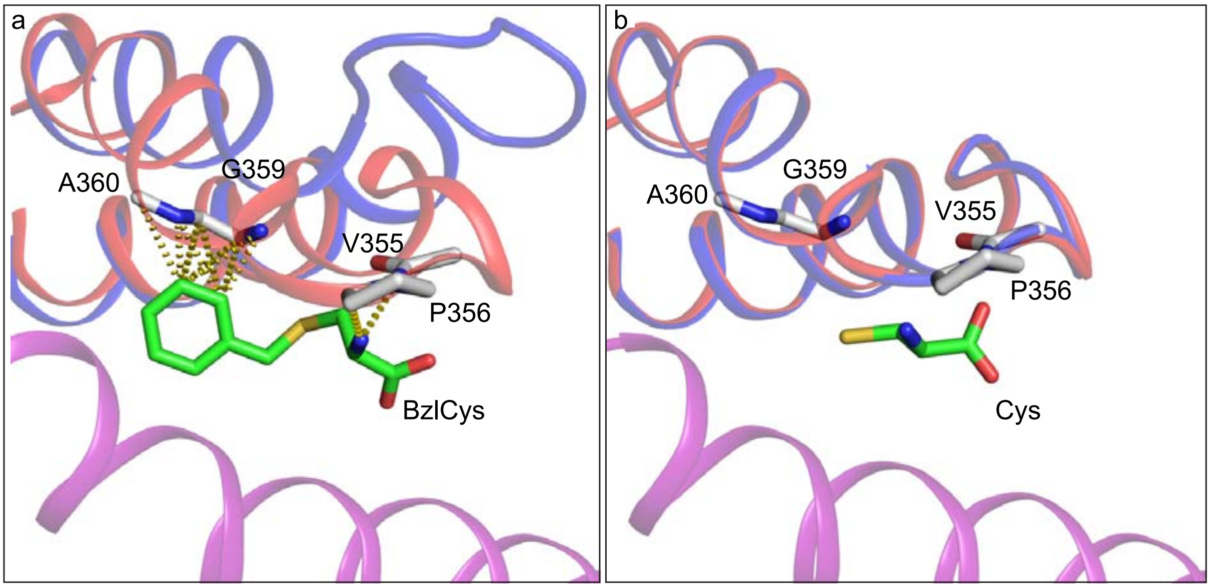

Supplementary Figure 9. **Benzylcysteine clashes with HP2 in the substrate-bound state.** (a) R397C-Glt_{Ph} Benzylcysteine bound structure and (b) R397C-Glt_{Ph} L-Cysteine bound structure, HP2 (blue) and TM8 (purple). Clashes with HP2 modelled in the closed conformation based on the Glt_{Ph} aspartate bound structure (in red) were identified with the Arpeggio program[4] and reveal that benzylcysteine, but not cysteine, clashes with residues in HP2 when it is closed down over the substrate binding site. Substrate/inhibitor are shown in green sticks and V355, P356, G359 and A360 (HP2) are shown as grey sticks. Clashes are indicated by yellow dashed lines.

Supplementary Table 1. The apparent number of Na^+ ions coupled to binding of amino acids to Glt_{Ph}-R397C protein and the apparent binding affinity (K_D) measured at 100 mM Na^+

	n	95 % confidence	K_D (μM)
Ser (15#)	1.7 ± 0.04	1.6-1.8	9 ± 1
Cys (16)	1.5 ± 0.04	1.5-1.6	31 ± 3
BCys (20)	0.8 ± 0.04	0.7-0.9	1000 ± 300
Gln (21)	1.1 ± 0.04	1.0-1.2	1800 ± 400

Total number of binding isotherms fitted globally to obtain *n*.

Supplementary Table 2. Primer sequences used for site-directed mutagenesis

ASCT1T458S	Forward	TGTGGACCGGTCCACCACGGTGG
	Reverse	ATCCAGTCCACAGCCAGGATCAG
ASCT1T459C	Forward	TGGACCGGACCTGCACGGTGGTGAATGTGG
	Reverse	TCACCACCGTGCAGGTCCGGTCCACAATCC
ASCT1T458S/T459C	Forward	TTGTGGACCGGTCCTGCACGGTGGTGAATGTGG
	Reverse	TTCACCGTGCAGGACCGGTCCACAATCCAG
ASCT2S481T/C482T	Forward	AGTGGACAGGACCACTACGGTCCTCAACGTGG
	Reverse	AGCCAGTCCACGGCCAAG
Glt_{Ph}R397C	Forward	ACATGGGATGTACGATGGTCAACG
	Reverse	CGTTGACCATCGTACATCCCATGT
Glt_{Ph}G396S/R397C	Forward	CTTAGACATGTCATGTACGATGGTCAACGTC
	Reverse	ATTGCGTCTATTCCAAGG

Supplementary Methods

Analysis of ligand binding data. The voltage sensitive amphipathic dye RH421 reports on Na^+ binding to purified Glt_{Ph} protein in detergent[3]. RH421 was used to follow Na^+ binding to Glt_{Ph}-R397C protein either alone or in reactions coupled to binding of amino acids. Isotherms of Na^+ binding in the absence of amino acids were fitted to the Hill equation, yielding $K_{D,Na}$ of 38 ± 3 mM and a Hill coefficient n_{Na} of 1.2 ± 0.1. Coupled ions and amino acid binding was probed by titrating amino acids to the Glt_{Ph}-R397C protein in the presence of 10 – 100 mM Na^+ ions. The obtained isotherms were analyzed to estimate an apparent number of Na^+ ions coupled to binding of the amino acids. Notably, titrations of Na^+ ions alone reveal significant ion binding in this concentration range. Thus, simple log-log plots of apparent ligand K_D-s as a function of Na^+ concentration underestimate coupling. Raw data were first normalized by the initial fluorescence of RH421 and plotted as fraction of fluorescence change (Supplementary Figure 7). The maximal fractional change varies between experiments as it depends strongly on the total amount of dye and detergent in the assay buffer. Nevertheless, we observed a general trend where the change is smaller at higher Na^+ concentrations, reflecting partial Na^+ binding to the protein prior to the start of the titration.

To take into account significant amino acid-independent Na^+ binding, the sets of binding isotherms obtained at variable Na^+ concentrations were fitted globally to a simplified binding equation:

$$Q = 1 + \frac{[Na^+]^{n_{Na}}}{{K_{D,Na}}^{n_{Na}}} + \frac{[A][Na^+]^n}{K_{D,a}}$$

$K_{D,a}$ is the combined dissociation constant for coupled Na^+ and ligand binding and n is the apparent number of coupled ions. Fraction of the protein bound to either Na^+ ions alone or to the ions and the amino acid is:

$$F_B = \frac{Q-1}{Q}$$

Fraction of the protein already bound to Na^+ ions before ligand addition is:

$$F_I = \frac{[Na^+]^{n_{Na}}}{{K_{D,Na}}^{n_{Na}} + [Na^+]^{n_{Na}}}$$

Fractional fluorescence change Y is fitted to an equation:

$$Y = Y_0 + \frac{\Delta Y}{1 + F_I \cdot \Delta Y} \cdot (F_B - F_I)$$

where Y_0 and ΔY are, respectively, baseline and the signal difference between fully bound and apo protein.

Notably, $K_{D,Na}$ and n_{Na} are, in principle, determined separately in Na^+ only titrations. However, we noted systematic improvements in the fits when we used slightly altered values. Thus we used $K_{D,Na}$ = 50 mM and n_{Na} = 1.1 for all datasets. $K_{D,a}$ and n were fitted globally for each ligand. Y_0 and ΔY were adjusted for each isotherm independently. The fits describe the data very well (Supplementary Figure 7) and yield apparent number of coupled ions n (Supplementary Table 1). We also fitted each individual isotherm to a standard Hill equation, plotted the apparent dissociation constants as functions of Na^+ concentrations and compared the values to those predicted using the parameters from the global fits (Supplementary Figure 8).

Comparison of these data to data previously obtained for the wild type Glt_{Ph} protein binding aspartate[3] shows drastic differences in the extent of coupling, with n value of 2.9 for coupled Na^+ and aspartate binding to Glt_{Ph} compared to n value of 1.7 for serine binding to Glt_{Ph}-R397C (Supplementary Figure 8). Collectively, these results suggest that one or more Na^+ ions bind tighter to R397C mutant than to the wild type protein and that affinity of the substrate amino acids for the transporter is less dependent on whether the ions are bound or not. Crystal structures suggest that the geometry of Na1 and Na3 sites are preserved in the R397C mutant; and Na2 site also looks intact in structures with closed HP2 loop, suggesting that the total number of Na^+ binding sites is not reduced. Tighter Na^+ binding and reduced coupling between ions and substrate has already been reported for mutants lacking arginine in position 397[5]. These results support the importance of the positive charge in position 397 in establishing intrinsically weak Na^+ binding that is strongly coupled to substrate binding.

Coupling is further reduced for glutamine and benzyl-cysteine. The most parsimonious explanation for this phenomenon compatible with crystal structures is that HP2 remains open when these ligands bind, and Na^+ does not bind to the Na2 site.

Supplementary References

1 Murshudov, G. N. *et al.* REFMAC5 for the refinement of macromolecular crystal structures. *Acta Crystallogr. D. Biol. Crystallogr.* **67**, 355-367, doi:10.1107/S0907444911001314 (2011).

2 Boudker, O., Ryan, R. M., Yernool, D., Shimamoto, K. & Gouaux, E. Coupling substrate and ion binding to extracellular gate of a sodium-dependent aspartate transporter. *Nature* **445**, 387-393 (2007).

3 Reyes, N., Oh, S. & Boudker, O. Binding thermodynamics of a glutamate transporter homolog. *Nat Struct Mol Biol* **20**, 634-640, doi:10.1038/nsmb.2548 (2013).

4 Jubb, H. C. *et al.* Arpeggio: A Web Server for Calculating and Visualising Interatomic Interactions in Protein Structures. *J. Mol. Biol.* **429**, 365-371, doi:10.1016/j.jmb.2016.12.004 (2017).

5 Focke, P. J., Annen, A. W. & Valiyaveetil, F. I. Engineering the glutamate transporter homologue GltPh using protein semisynthesis. *Biochemistry (Mosc).* **54**, 1694-1702, doi:10.1021/bi501477y (2015).
